# Mediating role of gestational weight gain in the relationship between socioeconomic status and preterm birth: a Chinese population-based study

**DOI:** 10.1186/s12889-024-19445-2

**Published:** 2024-07-15

**Authors:** Xiaomei Xiang, Yan Huang, Ziping Wang, Zongkai Li, Shaonong Dang

**Affiliations:** 1 School of Public Health, Xi’an Jiaotong University, Xi’an, 710061 China; 2 Xi’an Maternal and Child Healthcare Hospital, Xi’an, China

**Keywords:** Socioeconomic status, Gestational weight gain, Preterm birth, Mediation analysis, Socioeconomic disparities, Pregnancy

## Abstract

**Background:**

The modifiable mechanisms underlying the association between socioeconomic status (SES) and preterm birth remain unclear. This study aimed to investigate the relationship between preterm birth and maternal SES or gestational weight gain (GWG), as well as the role of GWG in mediating SES disparities in preterm birth.

**Methods:**

Data was from a hospital-based sub-study of physical growth and development survey for Chinese newborns with various gestational ages. Singleton newborns aged from 24 to 42weeks’ gestation and their mothers were included. Using information from maternal questionnaire, a composite SES was constructed with parental education and family annual income. GWG as mediator was calculated by deducting pre-pregnancy weight from maternal weight at delivery. Logistic regression model was adopted to investigate the association of preterm birth with SES or GWG. Causal mediation analysis was performed to measure mediating effect of GWG on the pathway from SES to preterm birth.

**Results:**

After controlling for potential confounders, risk of preterm birth was reduced by 12.4% (OR = 0.876, 95%CI:0.855–0.879) for per one-kilogram increase of GWG, and risk of preterm birth was reduced by 24% (OR = 0.760, 95%CI: 0.717–0.806) for per one-unit increase of SES score. Mediation analysis supported a significant association between higher SES and decreased risk of preterm partly through higher GWG, in which estimated proportion mediated by GWG was 13.04% (95%CI: 11.89–16.25). GWG also played a significant role as a mediator when socioeconomic status was indicated by maternal education, paternal education or family income. GWG mediated approximately 11.03% (95% CI: 8.56–18.25) of the total effect of SES on very preterm birth, which was greater than that for moderate preterm birth (6.72%, 95%CI: 2.72–31.52) and late preterm birth (9.04%, 95%CI: 5.24–24.04). A series of sensitive analysis confirmed the robustness of association of interest.

**Conclusion:**

Increased GWG and higher socioeconomic status are strongly associated with a lower risk of preterm birth. GWG mediates socioeconomic disparities in preterm birth, most notably in very preterm birth. Understanding this mechanism will aid in the development of interventions and policy for maternal and child health care.

**Supplementary Information:**

The online version contains supplementary material available at 10.1186/s12889-024-19445-2.

## Introduction

Preterm is defined as babies born alive before 37 weeks of pregnancy are completed. Premature newborns are often accompanied by respiratory system abnormalities, metabolic abnormalities, and other complications, which is a leading cause of under-five childhood deaths and has serious negative health, educational, and social outcomes [1]. In China, overall preterm birth rate increased from 5.9% in 2012 to 6.4% in 2018, with a significant 6.4% increase in singleton pregnancies and a 12.4% increase in multiple pregnancies [2]. Understanding determinants of preterm birth, as well as how to reduce dipartites in preterm birth, has a clear impact on maternal and child health. According to the hierarchically conceptual frame [3], socioeconomic status (SES) of pregnant women may be key because it is a distal determinant for the causal link of preterm birth, and changes in it may influence intermediate or even proximate determinants, thereby influencing the occurrence of preterm birth. Improving maternal SES would benefit more women and their families. SES is a comprehensive measure for social inequality, and typically measured by income, education, or occupation. SES individual dimensions, especially education [4, 5] or composite SES index were found to be closely related to adverse pregnancy outcomes [6–8].

Socioeconomic disadvantage does not directly increase risk of preterm birth, but rather through intermediary variables (mediators) that may sit on the mediating pathways linking maternal SES and preterm birth. Maternal lifestyle, mental health, and health care have been investigated as potential mediators of the pathway from maternal SES to preterm birth [9], but there is insufficient evidence on mediating effect of gestational weight gain (GWG). GWG is an important marker of maternal nutrition and fetal intrauterine growth. Low GWG was thought to be a risk factor for preterm birth [10, 11] but such link differed across cultures and ethnicities [12]. As a modifiable factor, GWG could imply possibility of public health measures. Intervention on GWG mediating pathway linking maternal SES and preterm birth could be a potentially effective way to reduce disparities in preterm birth. However, few studies have measured mediation effect via GWG. One study found a significant indirect effect of maternal SES on preterm birth via BMI and GWG together [13], and two other studies presented 5 or 7% mediating effect of GWG or BMI but did not estimate the statistical significance of such proportion [14, 15]. Therefore, a comprehensive assessment on GWG’s mediation role is required. This study used the hospital-based data to examine association of preterm birth with maternal SES or GWG, as well as mediating effect of GWG on pathway from maternal SES to preterm birth in Chinese.

## Methods

### Data and participants

Data was from Xi’an population-based study which was one part of physical growth and development survey for Chinese newborns with different gestational ages by the Ministry of Health of China. The design of the survey was described briefly here. Total twenty-one major hospitals with obstetrics department in Xi’an of China were selected to participant in this survey between July 2015 and August 2017. Participants were singleton newborns aged from 24 to 42 weeks’ gestation and their mothers. Mothers of newborns aged 18 to 40 years who had lived in Xi’an for more than 2 years were included, but those who smoked, abused alcohol or drug dependence 3 months before or during pregnancy, took adrenal cortical hormone or other immunosuppressive drugs for more than one month during pregnancy, and had severe anemia, diabetes, hyperthyroidism or hypothyroidism, cardiac and renal dysfunction, and chronic hypertension were excluded. The newborns were excluded when they were born to artificial pregnancy and had serious congenital malformation, limb deformity, fetal edema or chromosomal abnormality at birth or had missing information of gestational age. Finally, a total of 3225 newborns and their mothers were included in Xi’an study. A standard questionnaire was used for collection of information on birth outcome, family background, prenatal examination and anthropometric measurement for newborns (S-questionnaire). The staff were trained before investigation and testing tools and investigation procedures were unified. All questionnaires were reviewed and checked after investigation and the qualified ones were coded and inputted into the standard database. This study was approved by the institution’s ethics committee. Written informed consent was obtained from the mothers or parents of the newborns. The present study included 3203 participants, with 22 participants (0.68%) excluded due to missing values on key covariates such as annual family income, paternal age, and paternal education (Figure S1).

### Measures

#### SES and its component

In the present study, parental education level and annual family income were used as the variables reflecting socioeconomic status of the participants [4, 5]. The information on parental education level were obtained by asking the question like “how about your education level?” with five options such as primary school and below, junior school, senior school, college graduate and postgraduate. Information on annual family income was obtained by asking the question like “how much was total income of your family in the previous year?” with six options (thousands of Yuan) as < 50, 50-99.99, 100-299.99, 300-499.99, 500–1000, above 1000. Due to sparse data for some options, family income was re-classified into five categories as < 50, 50-, 100-, 300- and 500-. Composite SES was established based on parental education level and family income. Maternal education level, paternal education level and family income categories were assigned separately from low (1) to high (5), and composite SES scores were calculated via the summation of values of three socioeconomic variables above with ranging from 3 to 15 points. Increasing SES score reflected higher socioeconomic status. Considering magnitude of the adjusted relative risk of preterm birth for each individual SES variable, a similar but weighted SES was constructed using such formula as: SES-weighted = (β_1_×maternal education + β_2_×paternal education + β_3_×family annual income) × (3 /sum of the β coefficients), in which β_1_, β_2_, and β_3_ denoted multiple logistic regression coefficients of each individual SES variable with preterm birth, respectively. This weighted score also ranged from 3 to 15 points and increasing SES-weighted score also reflected higher socioeconomic status, which was used for sensitive analysis in the present study. Further, SES was dichotomized into low SES group (SES score < mean SES) and high SES group (SES score ≥ mean SES).

#### Gestational weight gain

According to the 2009 IOM standards, pre-pregnancy BMI was divided into four categories: underweight (18.5 kg/m2), normal weight (18.5–24.9 kg/m2), overweight (25–29.9 kg/m2), and obese (30 kg/m2). GWG was calculated by subtracting the pre-pregnancy weight from the maternal weight at delivery. According to the 2009 IOM Guidelines, the recommended GWG for underweight is 12.5–18 kg, 11.5–16 kg for normal weight, 7–11.5 kg for overweight, and 5–9 kg for obese [16]. Furthermore, inadequate GWG was defined as GWG less than the IOM Guidelines, otherwise, adequate GWG in the present study.

#### Preterm birth

Gestational age was determined according to mother’s last menstrual period (LMP) and the ultrasonic examination during early pregnancy. When LMP was not clear, ultrasonic examination was used to determine gestational age. Preterm birth refers to delivery before 37 weeks of gestation and it was further categorized into three subtypes in the present study: very preterm birth (gestational age < 32 weeks), moderate preterm birth (32 weeks ≤ gestational age < 34 weeks) and late preterm birth (34 weeks ≤ gestational age < 37 weeks). This classification was slightly different from traditional classification system [17]. Due to sparse number of extremely preterm, we combined the newborns with less than 28 weeks of gestation and those with 28 to less than 32 weeks of gestation as very preterm group.

### Conceptual model and covariates

Current literature showed potential association between socioeconomic status, GWG, and preterm birth [5–8, 11, 12]. So a conceptual model based on counterfactual frame was established to depict a possible causal relationship between SES and preterm birth via GWG considering potential confounders as Figure S2, in which SES was regarded to not only have direct effect on preterm birth but also has indirect effect via GWG, which acted as a mediator. Meanwhile, some covariates influenced socioeconomic status, GWG, or preterm birth, potentially confounding the above-mentioned relationship. This conceptual model is of interest because the mediated path of GWG has not been clearly identified. The covariates considered in the model included newborn gender (male and female), mode of delivery (vaginal delivery, caesarean section), ascertainment of gestational age, obstetric complications (yes or no), pre-pregnancy BMI, paternal age (years) (< 25, 25-, 30-, ≥ 35), gravidity (1, 2, 3 or ≥ 4 times), and parity (0–1 or ≥ 2 times), which were found related to preterm birth or SES in the previous studies [18]. The obstetric complications referred to anemia, pre-eclampsia, eclampsia, gestational hypertension, gestational diabetes, intrauterine infection, placenta previa, abruptio placentae, premature rupture of membranes, and fetal distress.

### Statistical analysis

Mean and standard deviation or numbers and percentages were employed to describe the characteristics of participants. Differences in SES, GWG and covariates among normal term, very preterm, moderate term and late term newborns were examined with χ^2^ test or ANOVA. Association of preterm birth with socioeconomic status or gestational weight gain was investigated by Logistic regression model and odds ratio and 95% CI were estimated. Considering impact of potential covariates, three adjusted models were established. Model 1 was adjusted for sex of newborns. Model 2 was adjusted further for delivery mode, ascertainment of gestational age, complications, gravidity, and parity based on model 1. Model 3 was adjusted additionally for pre-pregnant BMI, maternal age, paternal age, maternal education, paternal education and family annual income. Joint analysis was conducted to explore joint effect of GWG and SES or individual components of SES by Logistic regression model adjusting for the covariates as needed. Further, the dominance analysis was used to rank the relative contribution of individual components of SES to preterm birth based on decomposition of the R-square in multivariable logistic regression models [19].

A counterfactual-based mediation analysis was conducted, using the *medeff* command in STATA software, to ascertain if and by how much GWG mediated the total effect of SES on preterm birth [20]. In this analysis, the covariates above were adjusted as possible confounders. Average causal mediation effect of GWG, average direct effect of SES and average total effect of SES were assessed and then the proportion mediated by GWG (95% CI) was estimated. After mediation analysis, a sensitivity analysis was conducted by using *medsens* command to examine how robust the results were to the violation of the sequential ignorability assumption [20]. Two kinds of mediation analysis were completed in the main analysis. Firstly, GWG-mediated relationship between composite SES or individual components of SES and preterm birth was examined and then GWG-mediated relationship between composite SES and each type of preterm birth were evaluated further. In these analyses, composite SES and GWG were regarded as continuous variable.

A series of sensitive analysis was conducted to observed robustness of the results of interest. Firstly, mediation analysis was repeated with the mediator GWG considered as a categorical variable (inadequate or adequate GWG) to see how much the results changed when the type of mediator GWG was changed. Secondly, in mediation analysis, the weighted SES was used instead of SES to examine how much the results changed when using alternative estimation of socioeconomic status. Thirdly, because pregnancy complication and excess GWG could have strong impact on preterm birth [12, 21], mediation analysis was restricted to the participants without pregnancy complication or excess GWG. Finally, mediation analysis was repeated when excluding any or all of four covariates as gravidity, parity, determining gestational age, and pre-pregnancy BMI because they might be on the pathway from SES to preterm birth.

All analysis was performed using STATA (version 15 STATA Corp LP) and a two-sided P value < 0.05 was considered statistically significant.

## Results

### Characteristics of participants

Table 1 reports the characteristic of participants by gestational age. Totally 3203 participants were covered with 1399 preterm births. In comparison to the normal term newborns, the preterm newborns were different significantly in socioeconomic status (*P* < 0.001), and the preterm newborns had lower composite SES score (9.85 vs. 8.52). For individual components of SES, the parents of preterm newborns had lower education level and they tended to complete senior school or below. Over 30% of preterm newborn families had an annual income of less than 50,000 Yuan, compared to 13.7% of normal term newborn families. The mothers with premature newborn were inclined to have less GWG compared those with mature newborn (13 vs. 15.72). GWG was inadequate for more than one-third of mothers with premature newborns (36.9%), and this Fig. (54.3%) became the highest for those with very preterm newborns.

**Table 1:** Characteristics in relation to gestational age

	Normal term ≥ 37 weeks (*n* = 1804)	Very preterm < 32 weeks (*n* = 363)	Moderate preterm 32–33 weeks (*n* = 306)	Late preterm 34–36 weeks (*n* = 730)	Total preterm < 37 weeks (*n* = 1399)	P*
*Socioeconomic status (SES)*						
Individual components of SES						
Maternal education level(%)						
Postgraduate	201(11.1)	14(3.8)	8(2.6)	34(4.7)	56(4.0)	< 0.001
College graduate	1234(68.4)	142(39.1)	137(44.8)	373(51.1)	652(46.6)	
Senior school	225(12.5)	108(29.8)	86(28.1)	167(22.9)	361(25.8)	
Junior school and below	144(8.0)	99(27.3)	75(24.5)	156(21.3)	330(23.6)	
Paternal education level (%)						
Postgraduate	212(11.8)	17(4.7)	10(3.3)	45(6.2)	72(5.1)	< 0.001
College graduate	1217(67.5)	149(41.1)	139(45.4)	372(50.9)	660(47.2)	
Senior school	257(14.2)	110(30.3)	87(28.4)	165(22.6)	362(25.9)	
Junior school and below	118(6.5)	87(23.9)	70(22.9)	148(20.3)	305(21.8)	
Family income (◊thousands, Yuan) (%)						
< 50	247(13.7)	121(33.3)	99(32.4)	209(28.6)	429(30.7)	< 0.001
50-	1013(56.2)	184(50.7)	141(46.1)	356(48.8)	681(48.7)	
100-	501(27.8)	57(15.7)	61(19.9)	154(21.1)	272(19.4)	
300-	43(2.4)	1(0.3)	5(1.6)	11(1.5)	17(1.2)	
Composite SES						
SES ($\bar x{\mkern 1mu} \pm {\mkern 1mu} {\text{s}}$)	9.85 ± 1.67	8.22 ± 2.16	8.38 ± 2.17	8.73 ± 2.19	8.52 ± 2.19	< 0.001
Weighted-SES ($\bar x{\mkern 1mu} \pm {\mkern 1mu} {\text{s}}$)	10.22 ± 1.72	8.52 ± 2.25	8.68 ± 2.24	9.06 ± 2.25	8.84 ± 2.26	< 0.001
SES group (%)						< 0.001
Low SES	1198(66.4)	314(86.5)	260(85.0)	579(79.3)	1153(82.4)	
High SES	606(33.6)	49(13.5)	46(15.0)	151(20.7)	246(17.6)	
*Gestational weight gain (GWG)*						
Mean GWG (Kg, $\bar x{\mkern 1mu} \pm {\mkern 1mu} {\text{s}}$)	15.72 ± 4.88	11.20 ± 4.96	12.81 ± 4.53	13.99 ± 4.68	13.00 ± 4.87	< 0.001
GWG group						< 0.001
Inadequate GWG	283(15.7)	197(54.3)	119(38.9)	200(27.4)	516(36.9)	
Adequate GWG	1521(84.3)	166(45.7)	187(61.9)	530(72.6)	883(63.1)	
*Characteristics from newborn and its parents*						
Sex of newborn (%)						0.001
Male	890(49.3)	211(58.1)	180(58.8)	383(52.5)	774(55.3)	
Female	914(50.7)	115(41.9)	126(41.2)	347(47.5)	625(44.7)	
Ascertainment of gestational age (%)						< 0.001
LMP	1517(84.1)	206(56.8)	149(48.7)	450(61.6)	805(57.5)	
LMP plus B-ultrasound	287(15.9)	157(43.2)	157(51.3)	280(38.4)	593(42.5)	
Delivery mode (%)						< 0.001
Vaginal	1657(91.9)	175(48.2)	111(36.3)	305(41.8)	591(42.2)	
Cesarean	147(8.1)	188(51.8)	195(63.7)	425(58.2)	880(57.8)	
Gravidity (%)						< 0.001
1	1021(56.6)	121(33.3)	109(35.6)	316(43.3)	546(39.0)	
2	466(25.8)	103(28.4)	92(30.1)	203(27.8)	398(28.4)	
3	200(11.1)	77(21.2)	61(19.9)	106(14.5)	244(17.5)	
≥ 4	117(6.5)	62(17.1)	44(14.4)	105(14.4)	211(15.1)	
Parity (%)						< 0.001
0–1	1376(76.2)	201(55.4)	175(57.2)	471(64.5)	847(60.6)	
≥ 2	428(24.8)	162(44.6)	131(42.8)	259(35.5)	552(39.4)	
Complications (%)**						< 0.001
No	1755(97.3)	114(31.4)	67(21.9)	212(29.0)	393(28.1)	
Yes	49(2.7)	249(68.6)	239(78.1)	518(71.0)	1006(71.9)	
Pre-pregnancy BMI (kg/m^2^)(%)						< 0.001
< 18.5	363(20.1)	54(14.9)	34(11.1)	119(16.3)	207(14.8)	
18.5–24.9	1312(72.7)	263(72.5)	235(73.4)	536(73.4)	1034(73.9)	
25-29.9	114(6.3)	39(10.7)	35()11.4	60(8.2)	134(9.6)	
≥ 30	15(0.8)	7(1.9)	2(0.65)	15(2.1)	24(1.7)	
Maternal age (years) (%)						< 0.001
< 25	168(9.3)	59(16.3)	45(14.7)	87(11.9)	191(13.7)	
25-	1008(55.9)	136(37.4)	131(42.8)	349(47.8)	616(44.0)	
30-	495(27.4)	109(30.0)	92(30.1)	199(27.3)	400(28.6)	
≥ 35	133(7.4)	59(16.3)	38(12.4)	95(13.0)	192(13.7)	
Paternal age (years) (%)						< 0.001
< 25	63(3.5)	26(7.2)	17(5.6)	39(5.3)	82(5.9)	
25-	679(37.6)	114(31.4)	108(35.3)	282(38.6)	504(36.0)	
30-	745(41.3)	121(33.3)	106(34.6)	250(34.3)	477(34.1)	
≥ 35	317(17.6)	102(28.1)	75(24.5)	159(21.8)	336(24.0)	

Note: LMP: last menstrual period. *Comparison among normal term, very preterm, moderate term and late term newborns based on χ^2^ test or ANOVA. ** Refer to obstetric complications as anemia, pre-eclampsia, eclampsia, gestational hypertension, gestational diabetes, intrauterine infection, placenta previa, abruptio placentae, premature rupture of membranes, and fetal distress

### Potential association of preterm birth with SES or GWG

Table 2 shows GWG significantly associated with preterm birth. After controlling for all potential confounders, the risk of preterm birth was reduced by 12.4% (OR = 0.876, 95%CI:0.855–0.879) with per one-kilogram increase of GWG. Compared with those with inadequate GWG, the mothers with adequate GWG had a 66.1% lower risk of preterm birth in their offspring (OR = 0.339, 95%CI: 0.262–0.438). Higher socioeconomic status also was related to lower risk of preterm birth, and the risk of preterm birth was reduced by 24% (OR = 0.760, 95%CI: 0.717–0.806) with per one-unit increase of SES score after controlling for all potential confounders. Compared with those with low SES, the mothers with high SES had a 45.2% lower risk of preterm birth in their offspring (OR = 0.548, 95%CI: 0.423–0.711). Each component of SES was also found associated significantly with preterm birth. With increasing education level of parents, the risk of preterm birth significantly deceased. Compared with those with junior school and below, the risk of preterm birth in offspring was reduced by 61.4% (OR = 0.386, 95%CI:0.226–0.659) for the mothers with postgraduate education and 45.4% (OR = 0.546, 95%CI:0.325–0.917) for the fathers with postgraduate education after adjusted for all covariates. Similarly, the risk of preterm birth significantly deceased with increasing family income.

**Table 2:** Association of preterm birth with socioeconomic status and gestational weight gain

		OR (95%CI)	
	Model 1	Model 2	Model 3
Gestational weight gain (GWG)			
GWG (continuous variable)	0.890(0.876–0.904)	0.869(0.848–0.890)	0.876(0.855–0.879)
GWG group			
Inadequate	1.00(Reference)	1.00(Reference)	1.00(Reference)
Adequate	0.320(0.271–0.378)	0.307(0.240–0.392)	0.339(0.262–0.438)
Socioeconomic status (SES)			
SES (continuous variable)	0.703(0.675–0.731)	0.761(0.718–0.807)	0.760(0.717–0.806)
SES group			
Low	1.00(Reference)	1.00(Reference)	1.00(Reference)
High	0.423(0.357–0.501)	0.551(0.425–0.714)	0.548(0.423–0.711)
Individual components of SES			
Maternal education level			
Junior school and below	1.00(Reference)	1.00(Reference)	1.00(Reference)
Senior school	1.051(0.777–1.411)	1.064(0.746–1.517)	1.059(0.742–1.551)
College graduate	0.477 (0.352–0.645)	0.588(0.410–0.844)	0.585(0.407–0.840)
Postgraduate	0.291(0.186–0.455)	0.389(0.229–0.660)	0.386(0.226–0.659)
Paternal education level			
Junior school and below	1.00(Reference)	1.00(Reference)	1.00(Reference)
Senior school	0.682(0.500–0.930)	0.743(0.517–1.069)	0.752(0.552–1.083)
College graduate	0.465(0.337–0.642)	0.568(0.389–0.828)	0.589(0.403–0.862)
Postgraduate	0.395(0.254–0.614)	0.540(0.370–0.886)	0.546(0.325–0.917)
Family income (◊thousands, Yuan)			
< 50	1.00(Reference)	1.00(Reference)	1.00(Reference)
50-	0.595(0.486–0.728)	0.620(0.488–0.786)	0.615(0.484–0.781)
100-	0.624(0.491–0.795)	0.594(0.445–0.791)	0.602(0.451–0.803)
300-	0.449(0.245–0.822)	0.365(0.175–0.761)	0.371(0.177–0.778)

Note: Logistic regression model was used to estimate OR and its 95% CI, and CI not covering 1.00 indicated statistical significance (*P* < 0.05). Model 1 was adjusted for sex of newborns, Model 2 was adjusted further for delivery mode, ascertainment of gestational age, complications, gravidity, and parity, and Model 3 was adjusted further for pre-pregnant BMI, maternal age, paternal age, maternal education, paternal education and family annual income. For SES, Model 3 was just further adjusted for maternal age, paternal age and pre-pregnant BMI.

### Joint impact of SES and GWG on preterm birth

Table 3 indicates that compared with those with low SES and inadequate GWG, the mothers with higher SES or adequate GWG tended to be lower risk of preterm birth in offspring controlling for potential confounders. Importantly, a combined association was observed as higher SES tended to further enhance the positive impact of adequate GWG. Compared to those who were in low SES and adequate GWG, the mothers in high SES and adequate GWG were at lower risk of preterm birth (OR = 0.20, 95%CI: 0.139–0.288). The similar results were also observed for each component of SES. With increasing maternal education level, the risk of preterm birth decreased significantly even though GWG was not adequate. In comparison to those with junior schooling and below and inadequate GWG, the mothers with postgraduate education and inadequate GWG had 70.6% lower risk of preterm birth in offspring (OR = 0.294, 95%CI: 0.102–0.853). Such odds ratio continued to decrease in the mothers with postgraduate education and adequate GWG (OR = 0.149, 95%CI: 0.065–0.345), which implied that higher maternal education level seemed to enhance the positive impact of adequate GWG. Similar outcomes were observed when the joint effects of GWG and paternal education or family annual income were considered. Further, the dominance analysis was performed to isolate the contribution of each component of SES to preterm birth (Table S1). Maternal education was the top contributor (3.89%, standardized contribution = 47.08%) among the three domains, while paternal education (3.26%) and family income (1.12%) ranked second and third, respectively, implying that parental education level contributed more than family income.

**Table 3:** Joint analysis of impact of socioeconomic status and gestational weight gain on preterm birth

	GWG	*n*	OR(95%CI)	
SES				
Low	Inadequate	628	1.00(Reference)	
High	Inadequate	171	0.545(0.324–0.917)*	
Low	Adequate	1723	0.302(0.227–0.401)**	
High	Adequate	681	0.200(0.139–0.288)**	
Individual components of SES				
Maternal education				
Junior school and below	Inadequate	180	1.00(Reference)	
Senior school	Inadequate	186	0.914(0.454–1.839)	
College graduate	Inadequate	375	0.505(0.264–0.966)*	
Postgraduate	Inadequate	58	0.294(0.102–0.853)*	
Junior school and below	Adequate	294	0.326(0.178–0.595)**	
Senior school	Adequate	400	0.235(0.128–0.429)**	
College graduate	Adequate	1511	0.183(0.100-0.337)**	
Postgraduate	Adequate	199	0.149(0.065–0.345)**	
Paternal education				
Junior school and below	Inadequate	160	1.00(Reference)	
Senior school	Inadequate	187	0.982(0.479–2.009)	
College graduate	Inadequate	398	0.539(0.272–1.067)	
Postgraduate	Inadequate	54	0.698(0.243–2.008)**	
Junior school and below	Adequate	263	0.338(0.178–0.644)**	
Senior school	Adequate	432	0.219(0.116–0.412)**	
College graduate	Adequate	1479	0.221(0.117–0.421)**	
Postgraduate	Adequate	230	0.179(0.078–0.412)**	
Family income (◊thousands, Yuan)				
< 50	Inadequate	225	1.00(Ref)	
50-	Inadequate	405	0.569(0.334–0.970)*	
100-	Inadequate	153	0.341(0.170–0.683)*	
300-	Inadequate	16	0.859(0.220–3.357)	
< 50	Adequate	451	0.265(0.156–0.450)**	
50-	Adequate	1289	0.190(0.117–0.309)**	
100-	Adequate	620	0.180(0.104–0.309)**	
300-	Adequate	44	0.166(0.054–0.503)**	

Note: Those analyses were adjusted for sex of newborns, delivery mode, ascertainment of gestational age, complications, gravidity, and parity, pre-pregnant BMI, maternal age, and paternal age. *P < 0.05, **P < 0.01

### Role of GWG in mediating the relationship between SES and preterm birth

**Fig. 1:**
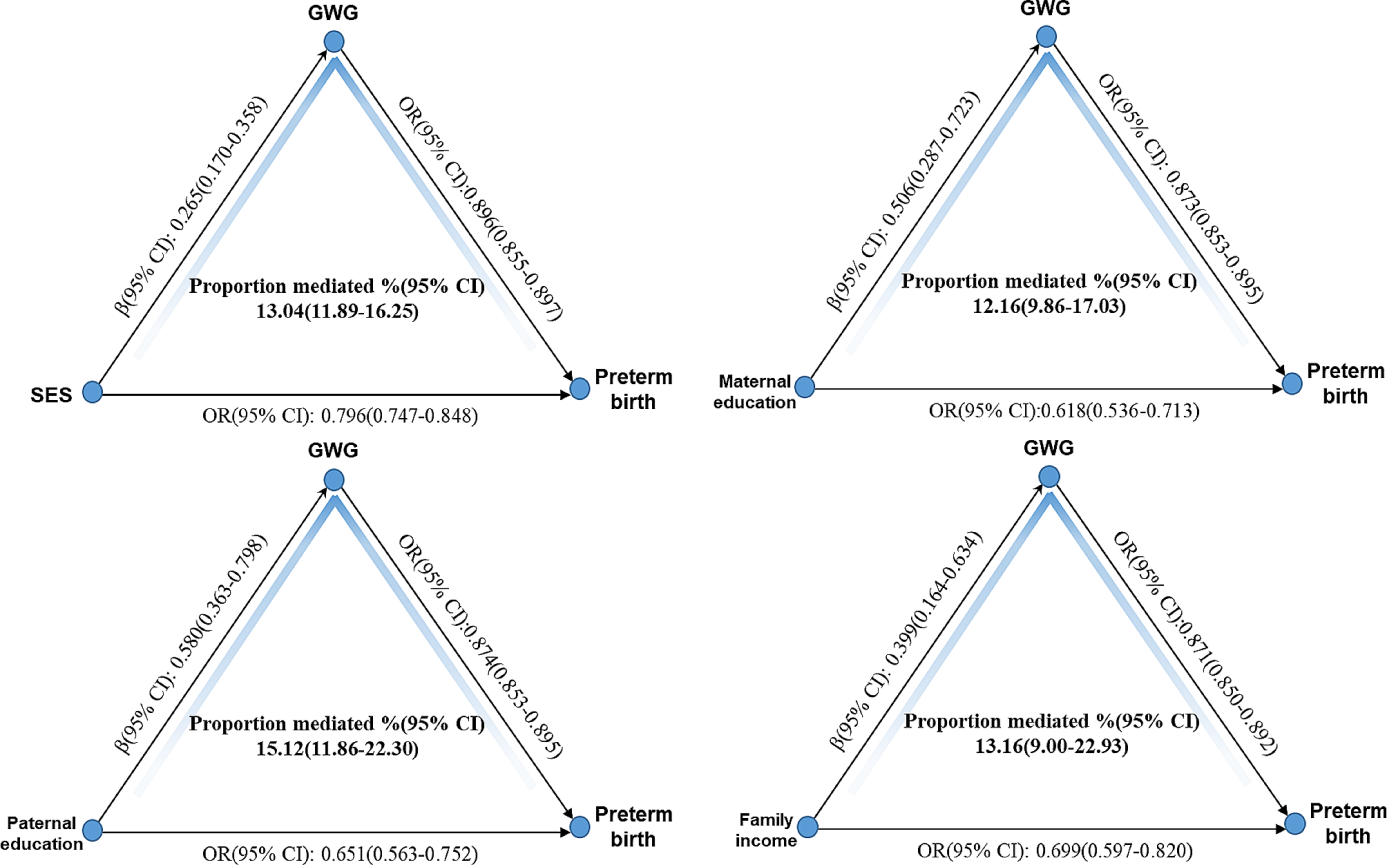
Association of SES and individual component with preterm birth via GWG. Note: Mediation analysis was adjusted for sex of newborns, delivery mode, ascertainment of gestational age, complications, gravidity, and parity, pre-pregnant BMI, maternal age, and paternal age

**Fig. 2:**
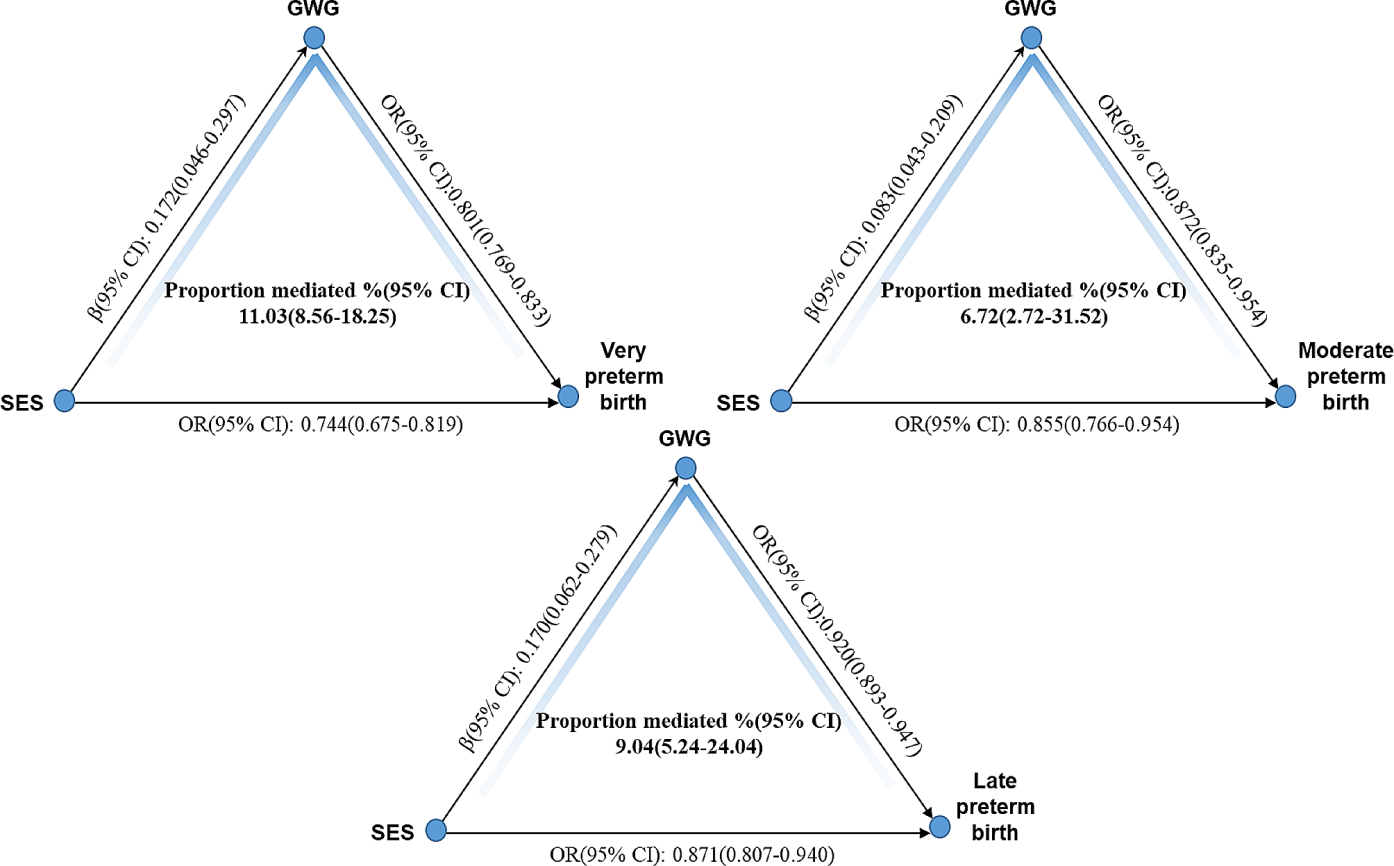
Association of SES with each type of preterm birth via GWG. Note: Mediation analysis was adjusted for sex of newborns, delivery mode, ascertainment of gestational age, complications, gravidity, and parity, pre-pregnant BMI, maternal age, and paternal age

Joint analysis pointed out that higher SES could further enhance the positive impact of adequate GWG. So, assuming GWG acting as a mediator, mediation analysis was conducted to explore impact of SES on preterm birth through GWG controlling for potential confounders. The proportion of the impact of composite SES or each component on preterm birth due to mediation is showed in Fig. 1. The overall effect of SES on preterm birth was decomposed into direct and indirect effect (mediation of GWG). The preterm birth was reduced directly by 20.4% for one-unit increase of SES (OR = 0.796, 95%CI: 0.747–0.848). SES increased GWG significantly (β = 0.265, 95%CI: 0.170–0.358), which reduced preterm birth by 10.4% (OR = 0.896, 95%CI: 0.855–0.897). So estimated proportion mediated by GWG was about 13.04% (95%CI: 11.89–16.25). The impact of each component of SES on preterm birth via GWG was also evaluated, and found that GWG acted significantly as mediator. GWG mediation accounted for 12.16% (95% CI: 9.86–17.03) of the total effect of maternal education on preterm birth, while this figure was around 15.12% (95% CI: 11.86–22.30) for paternal education. The proportion mediated by GWG was about 13.16% (95%CI: 9.00-22.93) when considering the effect of family income on preterm birth. In Fig. 2, the association of SES with subtype of preterm birth mediated by GWG was further evaluated. About 11.03% (95%CI: 8.56–18.25) of the total effect of SES on very preterm birth was mediated by GWG, which was larger than the proportion mediated by GWG for moderate preterm birth (6.72%, 95%CI: 2.72–31.52) and for late preterm birth (9.04%, 95%CI: 5.24–24.04).

### Sensitivity analysis

After analysis on association between SES and preterm birth mediated by GWG, a sensitivity analysis was conducted to examine robustness of the results to the violation of the sequential ignorability assumption. Figure S3 shows that the point estimate of the average mediation effect to be zero, the sensitivity parameter (ρ) was approximately 0.40, implying to some extent that possibly greater confounding effect would be needed to reverse such mediation effect. When mediation analysis was repeated when the mediator GWG was regarded as categorical variable (inadequate or adequate GWG), the results of interest were similar to those in the main analysis but effect size of mediation of GWG seemed to increase. For example, SES reduced risk of preterm birth significantly by reducing risk of inadequate GWG, and proportion mediated by inadequate GWG was about 15.04% (95%CI: 13.70-18.41) (Figure S4). When the weighted SES was used instead of SES in the mediation analysis, the mediation effect of GWG or inadequate GWG remained. The mediated proportion was about 13.10% (95%CI: 12.00-16.19) when GWG as mediator and 15.27% (95%CI: 13.93–18.45) when inadequate GWG as mediator (Figure S5). When the mediation analysis was restricted to the participants without pregnancy complication, the proportion mediated by GWG decreased to 9.85% (95%CI: 9.31–11.28) which was smaller than that in main analysis. The mediation effect of GWG did not change much for the participants without excess GWG (11.22%, 95%CI: 10.41–13.83) (Figure S6). When the mediation analysis was repeated after excluding each covariate as gravidity, parity, ascertainment of gestational age, pre-pregnant BMI, or all of them, the mediation effect remained regardless of whether the mediator was continuous or categorical GWG (Table S2).

## Discussion

The present study filled in an evidence gap that GWG significantly mediated socioeconomic disparities in preterm birth regardless of whether socioeconomic status was indicated using composite SES or individual components of SES among Chinese population. Additionally, the proportion mediated by GWG varied with degree of preterm birth and GWG had a greater mediation impact for very preterm birth compared to moderate and late preterm birth. Such findings suggest that modifying socioeconomic determinants to address inequalities in preterm birth could be alongside action on mediating pathways of GWG.

Previous studies proposed mechanisms underlying relations between SES and birth outcomes [6, 8, 22, 23], but the empirical evidence indicating maternal weight or GWG as a mediator through which SES influences preterm birth is sparse. Clayborne et al. found a significant association between lower neighborhood SES and increased risk of preterm birth through BMI and GWG in tandem, which was serial mediation [13]. A study showed that low pre-pregnancy BMI seemed to mediate effects of socioeconomic disadvantage on preterm birth but not significantly in statistic [7]. Unfortunately, the proportion mediated by GWG in these studies is unclear. More importantly, there has been not any study to assess GWG mediating impact of composite SES on preterm birth. The present study estimated a proportion mediated by GWG for impact of composite SES on preterm birth, which was about 13.04% among Chinese. Even using weighted SES, the mediated proportion remained 13.10%, suggesting robustness of mediation effect of GWG. Although there were not comparable studies, this estimated proportion demonstrated that residual effect of SES on preterm could be explained partly by GWG, and at least more than 10% of total effect of SES on preterm birth could be attributed to GWG in Chinese. It implies that women with higher SES were more likely to be of higher GWG, which in turn decreased risk of preterm birth. The joint analysis also promoted that the higher SES was inclined to further enhance the positive impact of adequate GWG. It means that the women with lower GWG who were in lower SES might be particularly susceptible to preterm birth, which represented a critical underserved target for intervention. Infants born preterm are at an increased risk of postnatal growth failure, cognitive impairments, and school difficulties [24, 25]. Therefore, in order to further reduce risk of preterm birth, one of feasible strategies is to both target the susceptible women with lower SES or lower GWG and improve maternal SES to increase maternal GWG appropriately.

Maternal education is the most important dimension used as indicator of SES [9] because it most strongly predicts health outcome of women and children [5]. Individual or household income and occupation are also frequently used to indicate socioeconomic status [6, 8]. All three variables or two of them are used to construct composite SES [4] according to accessibility of obtaining these variables in different studies. Therefore, it is difficult to compare directly composite SES across studies. Given that the importance of generally measuring a wider range of social factors in health research [26], a composite SES was constructed covering paternal education besides maternal education and family annual income in the present study because the role of father has recently received increased attention in biomedical research [27]. Paternal education was included so that this composite SES could reflect socioeconomic status of whole family. For comparing to previous studies, the present study further examined mediating effect of GWG for each component of composited SES. GWG accounted for 12.16% of total effect of increasing maternal education on reduced risk of preterm, which was higher than 4–5% in Danish study [14]. Another study in Dutch did not assess GWG mediation but found 7% of proportion mediated by BMI [15]. Such difference in mediated proportion could be attributed partly to different population and measures of maternal education. Paternal factors have been regarded to impact birth outcomes and father’s low education attainment explained a notable proportion of the disparity in preterm birth [28, 29]. A new finding was that not only increasing paternal education was associated significantly with reduced risk of preterm birth but also GWG played a partly mediating role in such association. And the proportion mediated by GWG for paternal education seemed little greater than that for maternal education (15.12% vs. 12.16%). In line with previous studies [30–32], the higher family income was also found associated with lower risk of preterm in Chinese, in which GWG’s mediating effect was similar to those in parental education. Hence, these analyses from individual component of SES further confirmed the fact of GWG playing a mediation role in association between SES and preterm birth. Dominance analysis also suggested that maternal and paternal education were main contributors to preterm birth compared to family income. Paternal education may be an important predictor of preterm birth, reflecting social and/or economic factors that are not measured by maternal education or family income [29, 33], and it should be prioritized in the strategy for improving maternal SES from a practical standpoint.

Interestingly, GWG appeared to have varying degrees of mediating effect between SES and subtypes of preterm birth. The contribution of GWG was most pronounced in SES disparities in very preterm birth, followed by late preterm birth. But the proportion mediated by GWG for moderate preterm birth appeared to decrease by 40% compared to very preterm birth (6.72% vs.11.13%). Similarly, the contribution of smoking was found varied over the entire range of preterm births in SES disparities in preterm birth [34]. Unfortunately, there were few studies addressing thoroughly this variation so that the reasons are not fully understood. A possible explanation is that very or late preterm birth might be more susceptible to socioeconomic factors or relevant scenarios in Chinese. A recent study in China found that the increase in preterm births was mainly due to an increase in the number of singletons born very preterm birth and multiples born late preterm birth but not moderate preterm births, which was partly due to such change of socio-demographical characteristics as an increased maternal age at delivery, proportion of mothers with complications, multiple pregnancies under implementation of the universal two child policy [2]. Such variation of GWG contribution suggests a clearly recognizable risk group, which could be a target for interventional attempts to reduce the incidence of preterm birth.

The mediation role of GWG was confirmed in the present study but it is necessary to address carefully this pathway acting effectively in the practice of maternal and child care. Individual socioeconomic status may create conditions that favor risk factors for preterm birth [22]. The women with lower SES were more likely to engage in health compromising behaviors as smoking, lower physical activity and poor fruit or vegetable intake [35], which in turn could be related to inappropriate GWG. Maternal educational attainment is less modifiable by life events, which is more important driver of GWG [22]. GWG below guidelines has been found associated with a higher risk of preterm birth [11, 12], which was possibly due to low GWG deducing macronutrient and micronutrient deficiency, or leading pathogenic status of mothers such as anemia and preeclampsia [36, 37]. Based on this important pathway, promoting maternal SES, particularly education level, is beneficial for increasing their awareness and ability of health care, which in turn improves pregnancy outcomes, while additional support should be provided to pregnant women who are most at risk of inappropriate GWG, thus providing women of all socioeconomic status equal opportunities to care for their own health.

The major strength of the study was to add new knowledge to mechanism of socioeconomic disparities in preterm birth. A new pathway was identified in which GWG significantly mediated the association between SES and preterm birth and quantitative estimates of proportion mediated by GWG was provided. Another strength was to determine variation of mediation role of GWG in the whole range of preterm birth. Moreover, sensitive analysis also confirmed robustness of mediation analysis. However, several limitations should be noted. First, due to nature of observational study, a real causal relationship could not be established completely. But reverse causation should not present because birth of infant must occur after exposure. Second, although lots of confounders were controlled in mediation analysis, there were still some unobserved or unknown confounders that could not be rooted out. But a sensitive analysis on violation of the sequential ignorability assumption suggested that greater confounding effect would be needed to reverse such mediation effect, meaning robust results. Further, after excluding some covariates the results of repeated mediation analysis seemed not be changed much. Third, occupation was not included in composite SES because of missing or inaccurate information of occupational classification, which could bring about potential misclassification error [6]. Fourth, the present study just examined mediation of GWG but not mediated interaction of SES and GWG because there was not interaction found between SES and GWG in the initial assessment. Fifth, the participants in the present study came from a single province of China, so the results may not be generalizable to other populations directly. Perspective study is required to further confirm this pathway.

## Conclusion

Increased GWG and higher socioeconomic status are strongly associated with a lower risk of preterm birth. GWG mediates partly socioeconomic disparities in preterm birth among Chinese. GWG’s mediation role varies across the preterm birth spectrum and it is most pronounced in SES disparities in very preterm birth. Such GWG mediation role supports efforts to better understand modifiable mechanisms in socioeconomic disparities in preterm birth, which will aid in the development of interventions and policies in the practice of maternal and child health care.

### Electronic supplementary material

Below is the link to the electronic supplementary material.

Supplementary Material 1

## Data Availability

The study data are available for academic purposes upon reasonable request from corresponding author.

## Abbreviations

SES: Socioeconomic status
GWG: Gestational week gain
IOM: Institute of Medicine
BMI: Body mass index
