# Supplementary material for: Mediating role of gestational weight gain in the relationship between socioeconomic status and preterm birth: a Chinese population-based study
Source: BMC Public Health. 2024 Jul 15;24:1886. doi: 10.1186/s12889-024-19445-2 (PMC11247897; doi:10.1186/s12889-024-19445-2)

**Supplementary documents**

Mediating role of gestational weight gain in the relationship between socioeconomic status and preterm birth: A Chinese population-based study

Xiaomei Xiang^1,2^, Yan Huang^1^, Ziping Wang^1^, Zongkai Li^1^, Shaonong Dang^1*^

^1^ School of Public Health, Xi’an Jiaotong University, Xi’an, China

^2^ Xi’an Maternal and Child Healthcare Hospital, Xi’an, China

**Contents**

Figure S1 Flow chart of the study design and selection of participants

Figure S2 Conceptual model of association between SES and preterm birth via GWG considering potential confounders

Figure S3 Sensitive analysis of mediation analysis

Figure S4 Association of SES and individual component with preterm birth via inadequate GWG

Figure S5 Association of SES-weighted with preterm birth via GWG or inadequate GWG

Figure S6 Association of SES with preterm birth via GWG when analysis restricted to the participants without pregnancy complications or excess GWG

Table S1 The contribution of each component of SES to preterm birth

Table S2 Proportion mediated by SES when excluding the covariates which possibly are on pathway from SES to preterm birth

S-questionnaire Questionnaire for survey on growth and development of Chinese newborn

Inclusion criteria

-Singleton newborns aged from 24 to 42 weeks’ gestation

-Mothers aged 18 to 40 years living in Xi'an for more than 2 years

Exclusion criteria

-Mothers who smoked, abused alcohol or drug dependence 3 months before or during pregnancy, and took immunosuppressive medicine

-Mothers with severe disease as severe anemia, diabetes, hyperthyroidism or hypothyroidism, cardiac and renal dysfunction, and chronic hypertension.

Xi’an hospital-based study

*A part of physical growth and development survey for Chinese newborns*

*with different gestational ages*

21 major hospitals with obstetrics department participating in the study

Eligible participants (n=3390)

Excluded participants for refusal to participate (n=165)

Included participants (n=3225)

Excluded participants for missing value

-Paternal age (n=2)

-Both paternal age and education (n=4)

-Maternal education (n=1)

-Annual family income (n=6)

-Pre-pregnant BMI (n=9)

Participants for final analysis (n=3203)

Figure S1 Flow chart of the study design and selection of participants


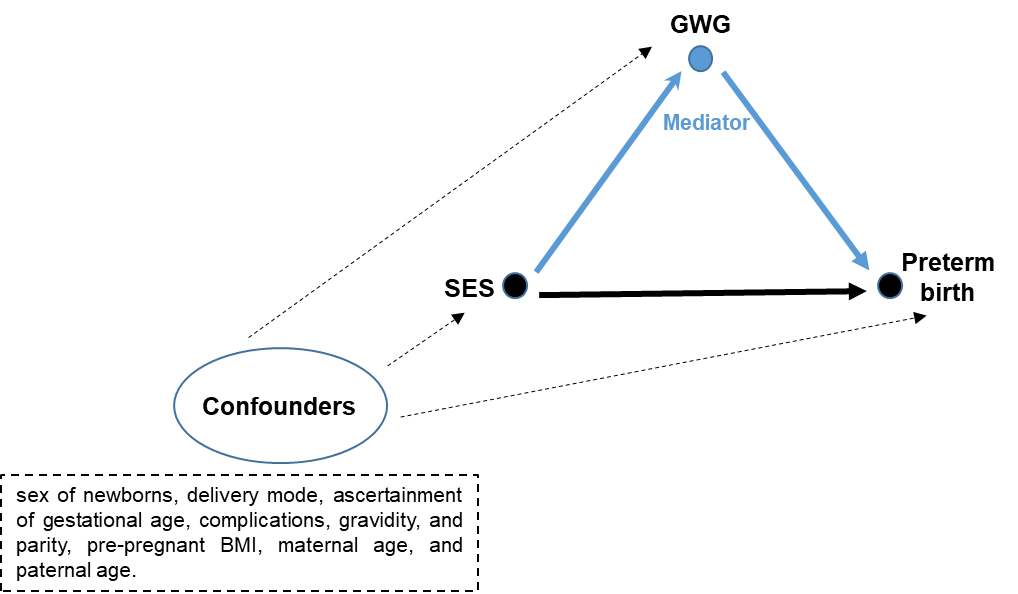


Figure S2 Conceptual model of association between SES and preterm birth via GWG considering potential confounders


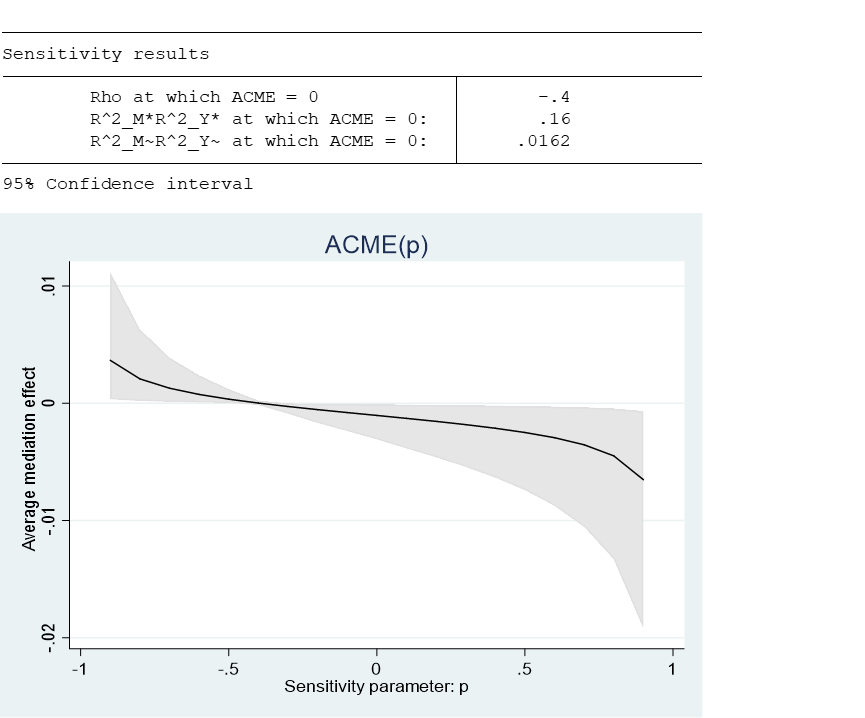


Figure S3 Sensitive analysis of mediation analysis


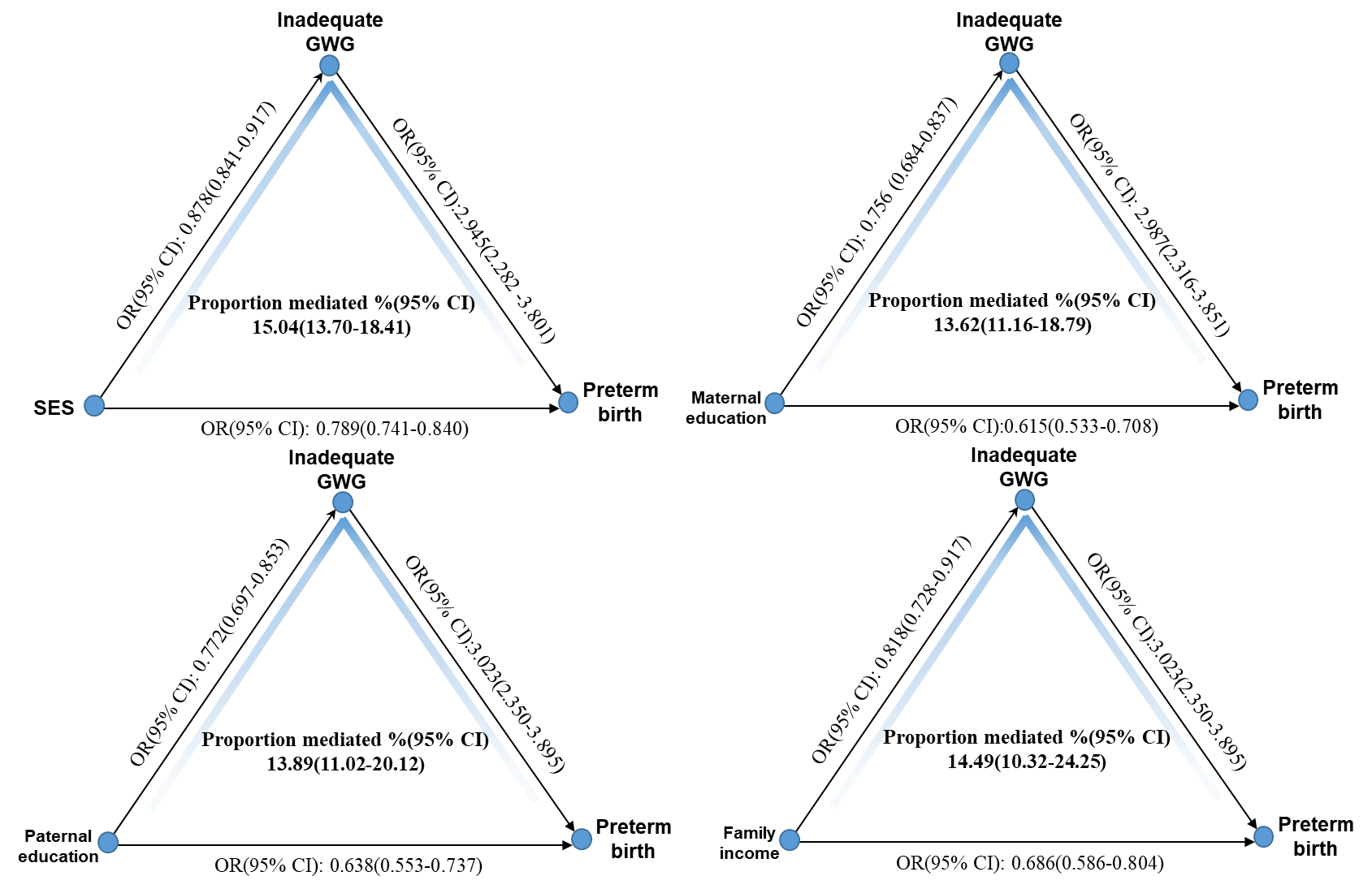


Figure S4 Association of SES and individual component with preterm birth via inadequate GWG

Note: Mediation analysis was adjusted for sex of newborns, delivery mode, ascertainment of gestational age, complications, gravidity, and parity, pre-pregnant BMI, maternal age, and paternal age.


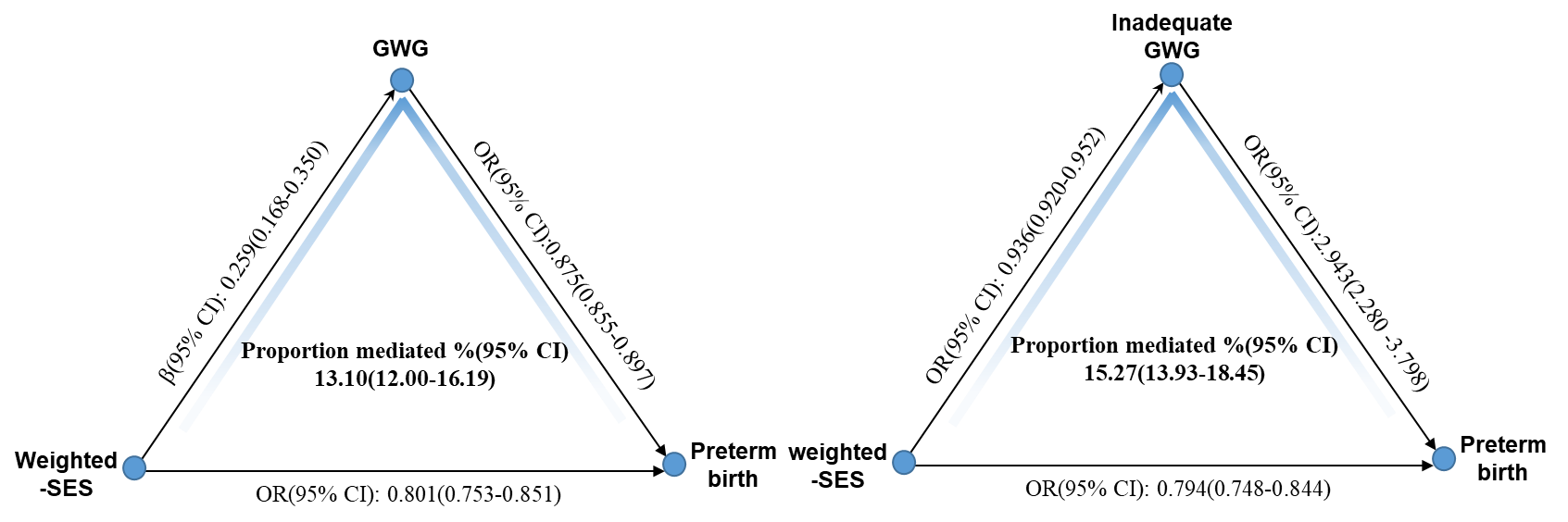


Figure S5 Association of SES-weighted with preterm birth via GWG or inadequate GWG

Note: Mediation analysis was adjusted for sex of newborns, delivery mode, ascertainment of gestational age, complications, gravidity, and parity, pre-pregnant BMI, maternal age, and paternal age.


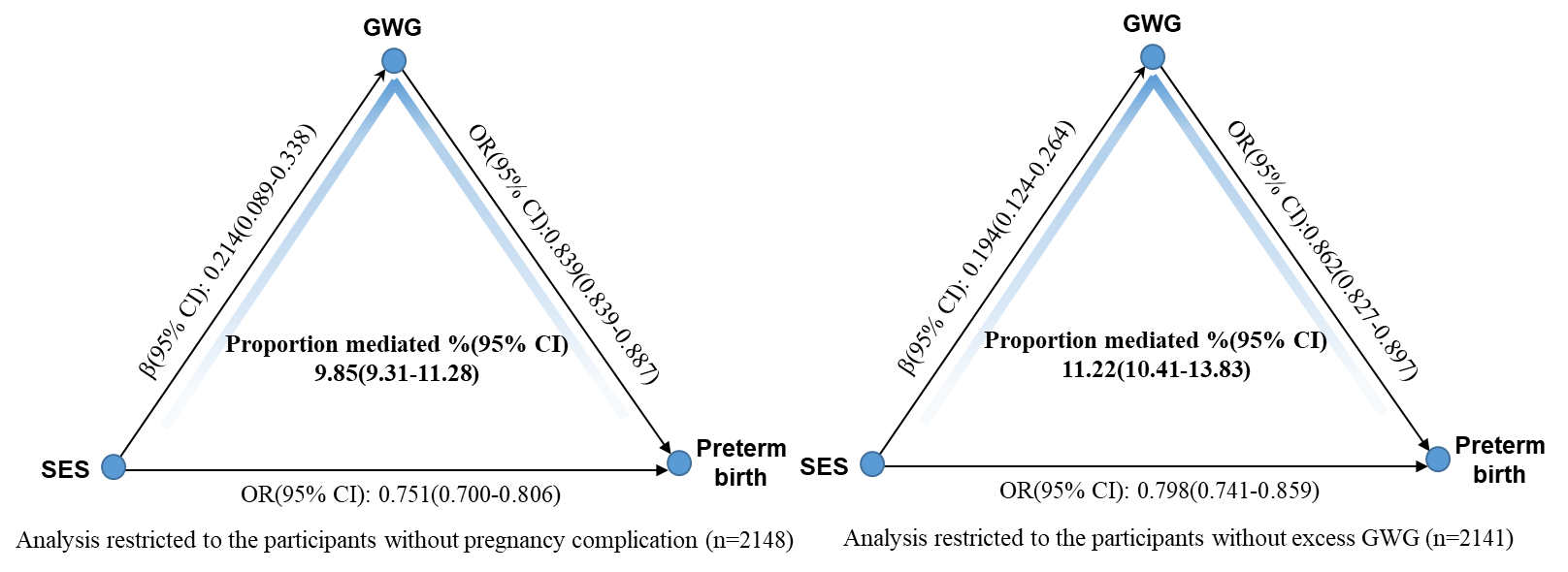


Figure S6 Association of SES with preterm birth via GWG when analysis restricted to the participants without pregnancy complications or excess GWG

Note: Mediation analysis was adjusted for sex of newborns, delivery mode, ascertainment of gestational age, complications, gravidity, and parity, pre-pregnant BMI, maternal age, and paternal age

Table S1 The contribution of each component of SES to preterm birth

| SES component | Domin. Stat.* | Standardized Domin. Stat.* | Ranking |
| --- | --- | --- | --- |
| Maternal education | 3.89 | 47.08 | 1 |
| Paternal education | 3.26 | 39.42 | 2 |
| Family income | 1.12 | 13.49 | 3 |

* The value was presented as percentage (%). SES: socioeconomic status.

Table S2 Proportion mediated by SES when excluding the covariates which possibly are on pathway from SES to preterm birth

|  | Proportion mediated by GWG (%) | CI |
| --- | --- | --- |
| Mediator: continuous GWG |  |  |
| Excluding gravidity | 13.04 | 12.13-15.48 |
| Excluding parity | 12.89 | 12.07-15.05 |
| Excluding ascertainment of gestational age | 12.63 | 12.03-15.83 |
| Excluding pre-pregnancy BMI | 13.13 | 12.03-15.83 |
| Excluding gravidity, parity, ascertainment of gestational age, and pre-pregnancy BMI | 11.56 | 10.97-12.79 |
| Adjusted for all covariates | 13.04 | 11.89-16.25 |
| Mediator: inadequate GWG |  |  |
| Excluding gravidity | 14.34 | 13.00-18.51 |
| Excluding parity | 14.06 | 12.88-17.59 |
| Excluding ascertainment of gestational age | 13.99 | 12.91-16.91 |
| Excluding pre-pregnancy BMI | 14.54 | 13.16-18.92 |
| Excluding gravidity, parity, ascertainment of gestational age, and pre-pregnancy BMI | 13.51 | 12.50-14.74 |
| Adjusted for all covariates | 15.04 | 13.70-18.41 |

Note: all covariates included sex of newborns, delivery mode, ascertainment of gestational age, complications, gravidity, and parity, pre-pregnant BMI, maternal age, and paternal age.

S-questionnaire


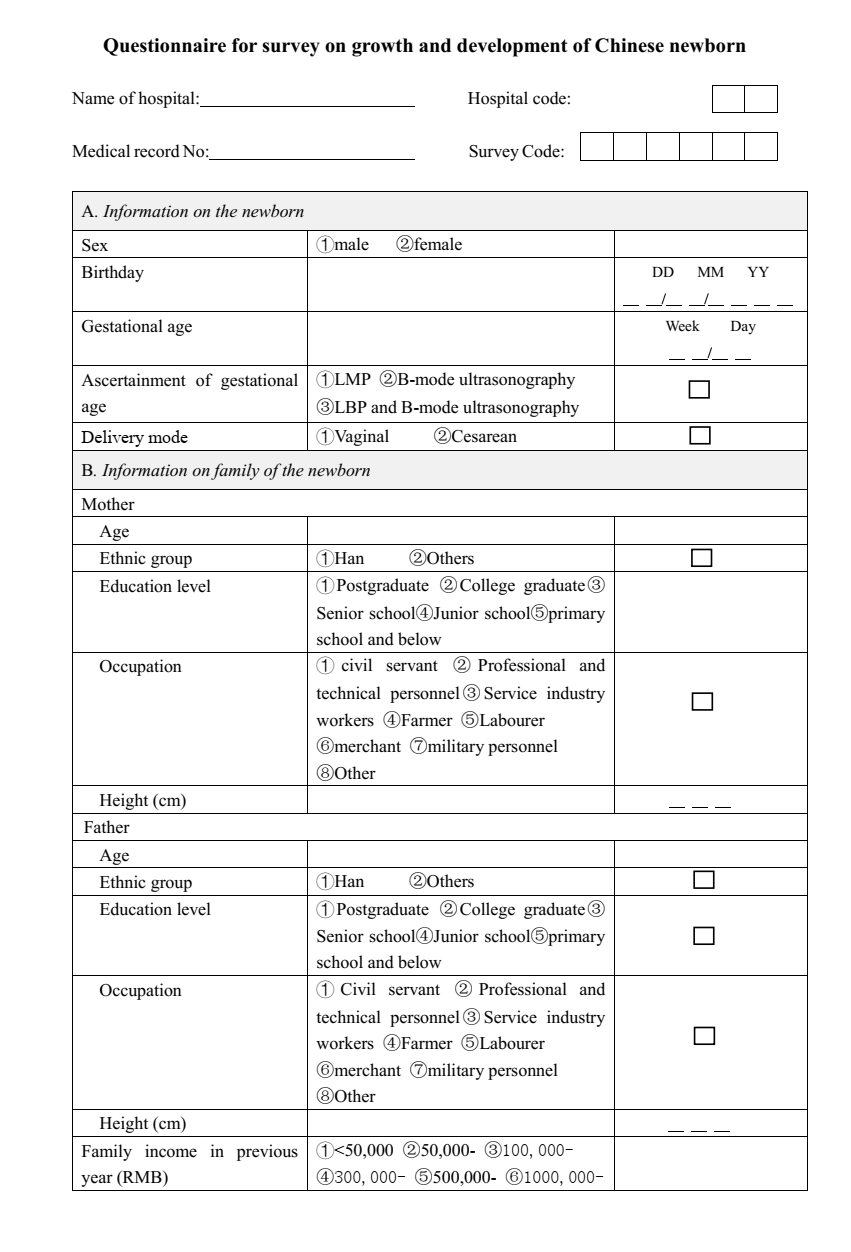


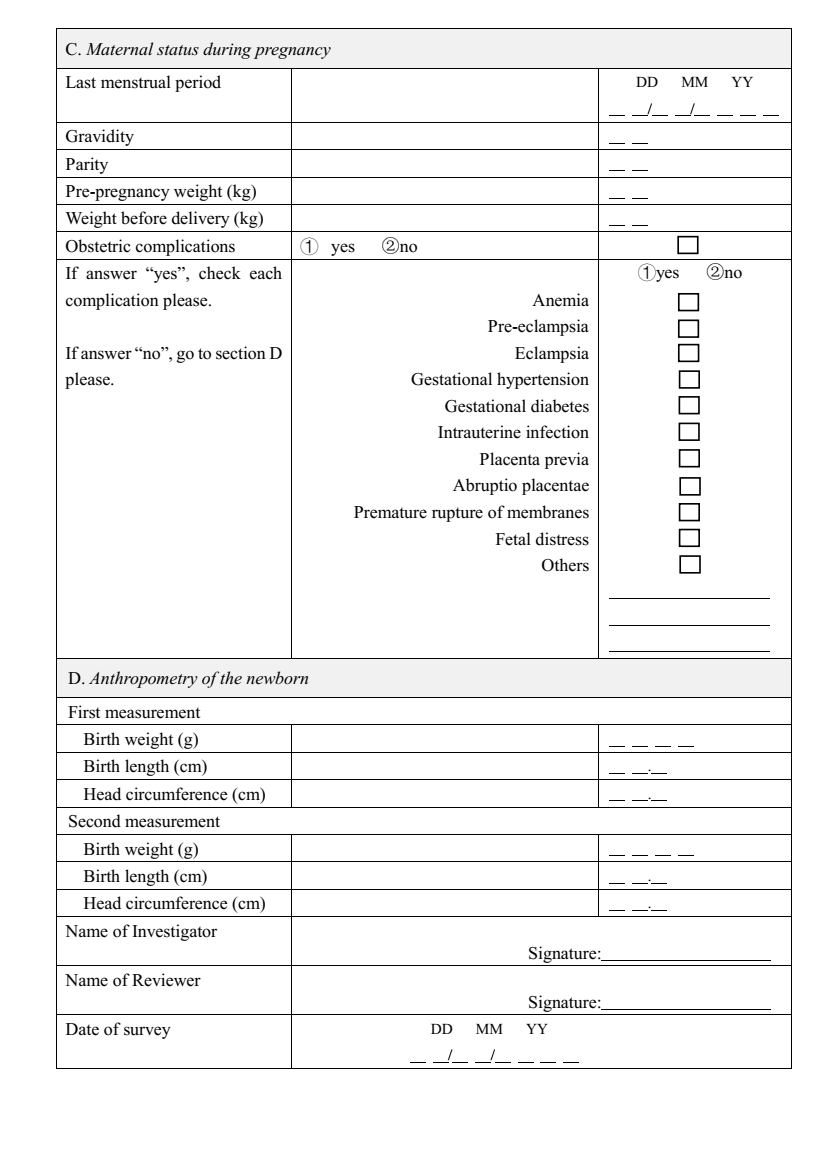

Supplement: Supplementary file 1 — Supplementary Material 1 [file 12889_2024_19445_MOESM1_ESM.docx]
